# Production of HIV Particles Is Regulated by Altering Sub-Cellular Localization and Dynamics of Rev Induced by Double-Strand RNA Binding Protein

**DOI:** 10.1371/journal.pone.0016686

**Published:** 2011-02-22

**Authors:** Silvio Urcuqui-Inchima, Claudia Patiño, Ximena Zapata, María Patricia García, José Arteaga, Christophe Chamot, Ajit Kumar, Danièle Hernandez-Verdun

**Affiliations:** 1 Grupo de Inmunoviología, Sede de Investigación Universitaria, Universidad de Antioquia, Medellín, Colombia; 2 Inmunología y Epidemiología Molecular, Universidad Industrial de Santander, Bucaramanga, Colombia; 3 Institut Jacques Monod, UMR 7592 CNRS/Université Paris-Diderot, Paris, France; 4 Department of Biochemistry and Molecular Biology, The George Washington University, Washington, D. C., United States of America; Virginia Polytechnic Institute and State University, United States of America

## Abstract

Human immunodeficiency virus (HIV)-1 encoded Rev is essential for export from the nucleus to the cytoplasm, of unspliced and singly spliced transcripts coding for structural and nonstructural viral proteins. This process is spatially and temporally coordinated resulting from the interactions between cellular and viral proteins. Here we examined the effects of the sub-cellular localization and dynamics of Rev on the efficiency of nucleocytoplasmic transport of HIV-1 Gag transcripts and virus particle production. Using confocal microscopy and fluorescence recovery after bleaching (FRAP), we report that NF90ctv, a cellular protein involved in Rev function, alters both the sub-cellular localization and dynamics of Rev *in vivo*, which drastically affects the accumulation of the viral protein p24. The CRM1–dependent nuclear export of Gag mRNA linked to the Rev Response Element (RRE) is dependent on specific domains of the NF90ctv protein. Taken together, our results demonstrate that the appropriate intracellular localization and dynamics of Rev could regulate Gag assembly and HIV-1 replication.

## Introduction

The nuclear factor 90 (NF90), a multifunctional double strand RNA-binding protein (DRBP), is involved in RNA splicing, mRNA-export and in antiviral response [1]–[8]. The NF90 family of proteins consists of diverse but closely related isoforms derived by alternative splicing of the interleukin enhancer binding factor 3 *ILF3*, gene [9]–[11]. The NF90 proteins share identical N-terminal and central regions but differ in their C-terminal domains. The N-terminal domain harbors sequences homologous to the NF45 and eIF2α proteins, as well as a nuclear export signal (NES), the central region contains a nuclear localization signal (NLS) and two double strand RNA-binding domains (DRBD1 and DRBD2), and the 70 amino acid C-terminal region is comprised of an arginine/glycine (RG) rich domain [12]. Among the NF90 family of proteins, NF90a/b is the smaller, (∼90 kDa), and NF110a/b the longer, (∼110kDa), protein. A four amino acid sequence (NVKQ insert) is present between DRBD1 and DRBD2 in the NF90b (NF90ctv) and in NF110b isoforms, whereas NF90a and NF110a lack this insert [12]. NF90 protein is normally localized in the nucleus/nucleolus. However, its concentration in the cytoplasmic compartment is increased in response to activation signals [12]. Recently it was demonstrated that phosphorylation of NF90 by the AKT serine/threonine kinase is necessary for export of NF90 to the cytoplasm where it interacts with the AU-rich element (ARE) present in the 3′-unstranslated region of interleukin-2 (IL-2) mRNA [13], [14].

Liao et al [15] reported that NF90 and the transcription co-activator, RNA helicase A (RHA), interact with highly structured RNAs such as the adenovirus RNAII. The affinity of NF90 proteins for various RNAs differs, dsRNA>virus associated (VA) RNAII>VA RNAI>ssRNA. NF90 associates with a nuclear export complex containing exportin 5 and Ran-GTP that participates in the nucleo/cytoplasmic shuttling of microRNAs [16]. As with RHA, NF90 participates in the replication cycle of several viruses; over-expression of NF90 in CD+/CXCR4+ human osteosarcoma cells was shown to induce the expression of IFN-dependent genes and block HIV-1 replication [17]. Isken and colleagues [4], [18] showed that the isoforms NF90/NFAR-1 complexes are essential for the replication of Hepatitis C virus (HCV). NF90 may negatively regulate influenza virus replication by interacting with the virus nucleoprotein, that is part of the polymerase complex essential for the initiation of viral replication [2]. The NF90/NFAR-1 complex is recruited by the replication machinery of Bovine viral diarrhea virus (BVDV), which positively regulates BVDV replication, a virus related to HCV [19]. Depletion of NFAR1/NFAR2 from murine fibroblasts rendered these cells dramatically susceptible to Vesicular stomatitis virus replication [3].

Viral proteins required to complete HIV-1 assembly are encoded by unspliced or partly spliced viral RNAs containing an untranslated 234 nucleotide-long RNA structure, known as Rev-responsive element (RRE) [20]. The RRE RNA contains a high-affinity binding site for the Rev protein, which allows shuttling of these transcripts from the nucleus to the cytoplasm in a CRM1-dependent manner; such RevRRE-CRM1 complexes can be disassembled by leptomycin B (LMB), an inhibitor of nuclear export [21], [22]. The Gag polyproteins are synthesized from an unspliced full-length viral genomic mRNA and their export from the nucleus requires host-cell derived and viral factors. Interestingly, HIV-1 Gag assembly appears to be regulated at an early step of nuclear export of singly spliced and unspliced mRNAs [23], [24]. In addition, targeting of HIV-1 matrix is regulated by Gag mRNA trafficking [25]. These results suggest that RNA export plays an essential role in Gag expression and viral assembly [26]. However, little is known about the regulation of viral RNA export and the significance of sub-cellular localization and dynamics of Rev in viral assembly.

An association between HIV-1 replication and NF90ctv was first proposed when over-expression of NF90ctv in human osteosarcoma cells resulted in resistance to HIV-1 replication [17]. Subsequently, it was demonstrated that NF90ctv can compete with HIV-1 Tat protein for transactivation response (TAR) RNA, leading to decreased HIV-1 transcription [5]. We also demonstrated that NF90ctv inhibits Rev-mediated RNA export by interacting with the Rev protein, suggesting a mechanism that depends on NF90 protein-RRE interaction [6].

In the present study we investigated the relationship between NF90ctv and the sub-cellular localization/dynamics of HIV Rev with HIV-1 particle production. We used confocal microscopy and fluorescence recovery after bleaching (FRAP), to show that NF90ctv and in particular, three of its functional domains are important in the recognition and export of Gag mRNA linked to RRE, and the process relies on sub-cellular localization and dynamics of Rev. Our results thus suggest a functional link between HIV-1 Gag assembly and HIV-1 budding with the subcellular localization and cellular mobility of Rev.

## Results

### Three functional domains of NF90ctv guide RRE-Gag mRNA export

**Figure 1:**
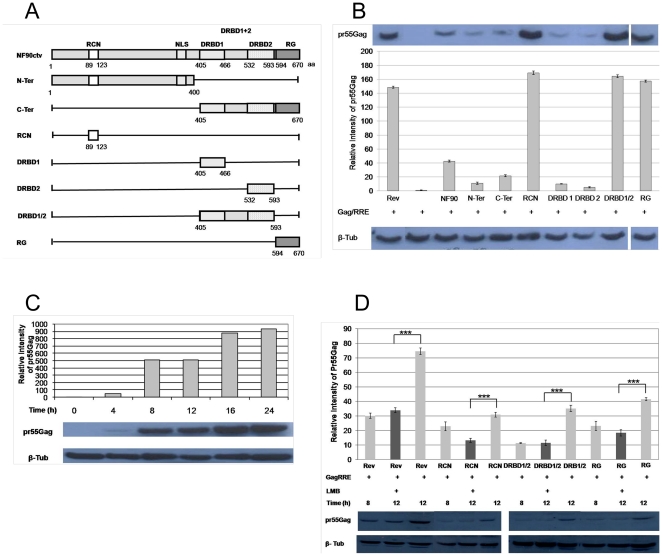
NF90ctv recognizes and exports transcripts linked to RRE. A) Schematic representation of full-length NF90ctv and of the various truncated forms used in this study. The main domains of NF90ctv and their positions are shown. B) Quantification and Western blot results demonstrating that NF90ctv and specifically, the RCN, DRBD1/2 and RG domains bind and export mRNA-Gag linked to RRE. HeLa cells were co-transfected with the construct pCMVGag2RRE in the presence of pNF90ctv-mRFP or each of its truncated forms. pRev-GFP was used as positive control. After 24 h Western blots were performed using pr55Gag antibodies. C) Kinetic and quantification of pr55Gag expression. HeLa cells were co-transfected with pRCN-mRFP in the presence of pCMVGag2RRE. At different times, the cells were lysed and Western blots were performed using pr55Gag antibodies. D) Quantification of the results obtained by Western blots demonstrates that RCN, DRBD1/2 and RG mRNA-Gag linked to RRE export is leptomycin-dependent. HeLa cells were cultured in six-well plates; three wells for each construct of interest were used: one well for leptomycin treatment and two wells as controls at 8 and 12 h. The cells were co-transfected with pRCN-mRFP or with pDRBD1/2-mRFP, with pRG-mRFP in the presence of pCMVGag2RRE. Based on the results shown in D, 8 h later the cells of one well were harvested; in the second well, leptomycin B was added and incubated for 4 h; finally, 12 h later, the cells of the second and the third wells were harvested. Western blots were performed on the cell pellets using pr55Gag antibodies. Each assay was repeated three times. The Western blots were quantified by densitometric scanning using ImageJ and normalized using loading controls (β-tubulin). The data shown are the means and standard errors of the mean of three independent experiments.

Previously we reported that specific domains of the NF90ctv protein influence the nuclear export function of Rev [6]. In these studies NF90ctv targeted Gag mRNA containing the RRE RNA for export to the cytoplasm and subsequent translation of Gag protein, suggesting that NF90ctv binds RRE, as does the HIV Rev protein. NF90ctv protein contains two DRBDs that strongly bind highly structured dsRNAs (such as the VA RNA II). We reasoned that the DRBDs alone or other region(s) of NF90ctv could recognize the RRE RNA. We constructed expression vectors for different NF90ctv protein domains (Figure 1A), co-transfected HeLa cells with the Gag-RRE vector (pCMVGag2RRE), and assessed pr55Gag expression by Western blots. First, the expression of pr55Gag in the presence of Rev was confirmed and served as positive control (Figure 1B). In the presence of NF90ctv, expression of pr55Gag was also observed (Figure 1B), however, the amount of pr55Gag was ∼4 fold lower than with Rev. This result shows that NF90ctv recognizes and facilitates nuclear export and translation of RRE-containing transcripts.

We next characterized the region(s) of NF90ctv that might recognize RRE RNA. Expression vectors for different regions of NF90ctv (Figure 1A) were co-transfect into HeLa cells along with pCMVGag2RRE, and the effects of the NF90ctv derivatives on RRE-mediated export were evaluated by Western blotting of pr55Gag. The NF90ctv protein domains (N-terminal amino acids 89–123 including the NES) designated as the region comprising the NES (RCN), the two DRBDs (DRBD1/2) between amino acids 405–593, and the RG-rich (RG) region (between amino acids 594–670), promoted higher pr55Gag expression as compared to other NF90ctv domains (Figure 1B); the expression level of pr55Gag was similar to that observed with Rev, used as positive control. Moreover, the DRBD1 and DRBD2 domains separately did not lead to pr55Gag expression, indicating that the two DRBP domains together are necessary for recognition of RRE and the shuttling p55Gag mRNA to the cytoplasm. These results suggest that three specific domains of NF90ctv, the N-terminal RCN, DRBD1/DRBD2 together, and the C-terminal RG promote Rev-RRE mediated nuclear export of Gag mRNA.

### The ability of NF90ctv to export RRE RNA is CRM1-dependent

The HIV-1 Rev protein is a product of fully spliced mRNA, which shuttles back to the nucleus where it interacts with RRE RNA structures present in intron-containing mRNAs; this interaction leads to the recruitment of exportin CRM1 and other cellular proteins for the export of Rev-RRE RNAs to the cytoplasm [27], [28]. We evaluated whether export of RRE RNA by the three NF90ctv protein domains is mediated by CRM1, using LMB to selectively block CRM1-mediated nuclear export [29]. The kinetics of Gag (pCMVGag2RRE) expression in the presence of the NF90ctv RCN, DRBD1/2 or the RG-rich domains at 4, 8, 12, 16 and 24 h following co-transfection of HeLa cells were examined. In the presence of RCN (Figure 1C), a weak expression of pr55Gag was detected at 4 h after co-transfection. The amount of pr55Gag increased with time, the highest level of expression was observed at 16 h (followed by stabilization at 24 h). Similar results were observed with the DRBD1/2 and RG proteins (results not shown). LMB was introduced at 8 h after co-transfection and incubated for additional 4 h. Thus, pr55Gag expression was quantified at 8 h and 12 h after transfection with or without LMB. The amount of pr55Gag produced at 8 h was similar to the amount detected after 12 h with LMB (Figure 1D), suggesting that treatment with LMB caused a decrease in pr55Gag mRNA export. A similar result was observed for Rev (control) at the same LMB concentration (Figure 1D); i.e. LMB blocks Rev-dependent nuclear export of Gag-RRE mRNA, as is the case with the three NF90ctv protein domains. These results suggest that the NF90ctv protein domains including the RCN, DRBD1/2 and RG regions recognize and export the HIV-1 intron-containing transcripts in a CRM1-dependent fashion.

### RCN, DRBD1/2 and the RG domains restrict late events of HIV-1 replication

**Figure 2:**
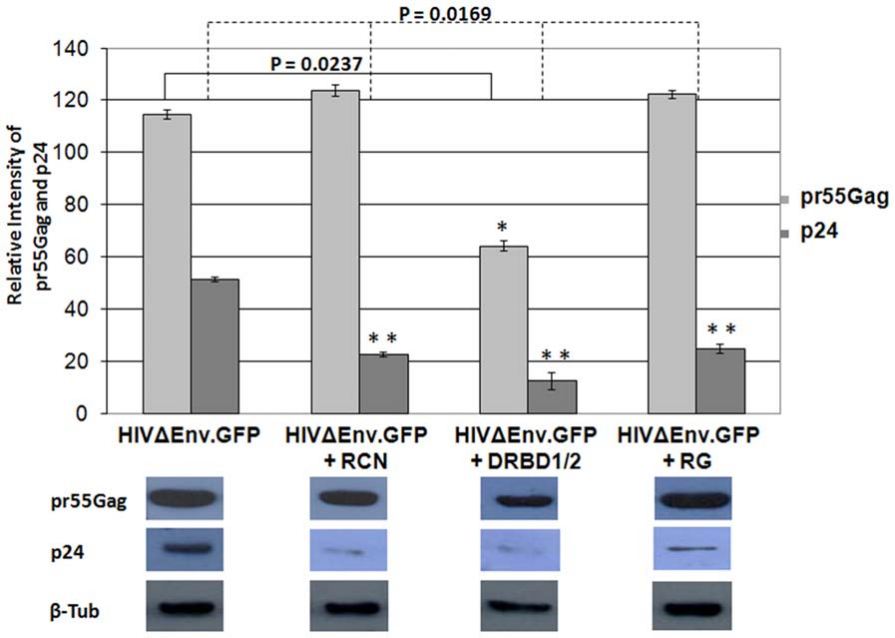
The RCN, DRBD1/2 and RG domains of NF90ctv inhibit accumulation of HIV-1 p24 in the cells. HeLa cells were co-trasnfected with each of the NF90ctv domains of interest in the presence of HIVΔEnv.GFP. After 24 h, Western blots were performed on the cell pellets using HIV-1IIIB pr55Gag antibodies. The quantification of the pr55Gag and p24 proteins was determined as described in Figure 1. The data presented are the means and standard errors of three independent experiments.

We next investigated whether the NF90ctv protein domains with the ability to bind the RRE-RNA are able to affect HIV-1 particle production. To approach this issue, we used the pHIV/Δenv-GFP construct whose virus progeny is not infectious. To test whether the three NF90ctv protein domains interfere with specific steps of HIV-1 life cycle, HeLa cells were co-transfected with pRCN-mRFP, pDRBD1/2-mRFP, or pRG-mRFP in the presence of pHIV/Δenv-GFP. Expression level of p24 was evaluated by Western blotting using an antibody that recognizes the pr55Gag polyprotein as well as p24. The results showed that over-expression of the NF90ctv protein domains strongly inhibited HIV-1 p24, compared with the expression of pHIV/Δenv-GFP alone (Figure 2). Inhibition of p24 expression was 2.5 fold in the presence of RCN and the RG domains of NF90ctv, whereas in the presence of DRBD1/2, the decrease was approximately 5 fold lower compared with the control (Figure 2). In contrast, the level of expression of pr55Gag in the presence of the NF90ctv protein domains remained unchanged compared to that of the control, indicating that the effect on HIV-1 particle production is not at the mRNA export step.

### The effects of NF90ctv on the amount of p24 protein expressed in pNL4-3 DNA transfected cells

**Figure 3:**
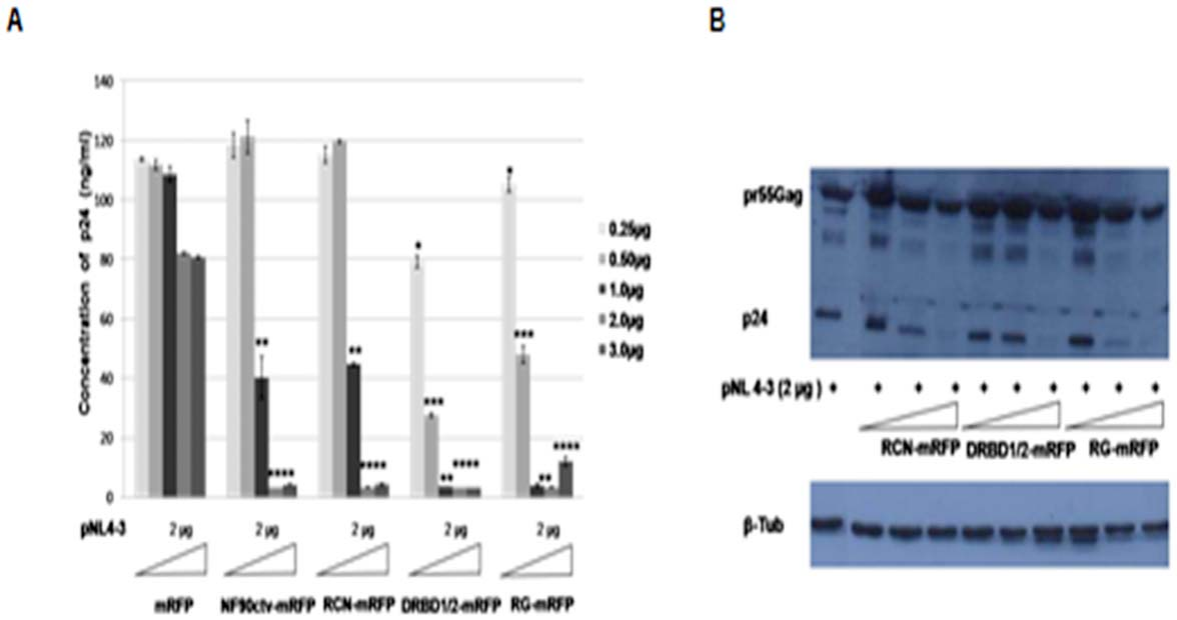
NF90ctv and three of its truncated forms block HIV-1 replication. A) ELISA results show that the effect of NF90ctv and three of its deletions on HIV-1 replication is dose-dependent. HeLa cells were co-transfected with pNL4-3 and with different concentrations of pNF90ctv-mRFP, pRCN-mRFP, DRBD1/2- mRFP or pRG-mRFP. After 24 h and 48 h the supernatants from the cell cultures were collected and assayed for HIV-1 p24 accumulation by ELISA. The cells used to obtain the results observed in A, were lysed and Western blots were performed on the cell pellets using HIV-1IIIB pr55Gag antibodies. B) NF90ctv and three of its deletions inhibit accumulation of p24 in the cells.

The possible effect of the NF90ctv protein domains on virus production using pNL4-3 infections was examined. HeLa cells were co-transfected with different amounts of plasmid DNA expressing NF90ctv, RCN, DRBD1/2 or RG in the presence of pNL4-3. An aliquot of the culture medium was removed at 24 and 48 h after transfection to measure the amounts of p24 secreted from infectious virus particles into the supernatants by ELISA or in cell lysates by Western blotting. In the absence of NF90ctv or its different deletion mutants, the control cells transfected with pNL-4-3 released virus into the supernatant (Figure 3A). In the presence of increasing concentrations of plasmid NF90ctv, RCN, DRBD1/2 or RG, the cells displayed a progressive reduction in the amount of virus released (p24), reaching undetectable levels of p24 production at higher concentrations (Figure 3A). Similar inhibition of p24 was observed in cell lysates (Figure 3B). Taken together these results suggest that expression of NF90ctv drastically curtails the amount of p24 expressed, suggesting inhibition of HIV-1 particle production. On the other hand, the amount of pr55Gag remained unchanged compared to the controls (Figure 3A), as was observed with pHIV/Δenv-GFP (Figure 2). Consequently, these effects of NF90ctv and its derivatives on pr55gag processing may be mediated by NF90ctv-directed sub-cellular redistribution and dynamics of Rev. To test this possibility, the effect of the three NF90ctv derivatives on the localization and dynamics of Rev was examined.

### NF90ctv-RRE-RNA Interaction alters HIV-1 Rev sub-cellular localization

**Figure 4:**
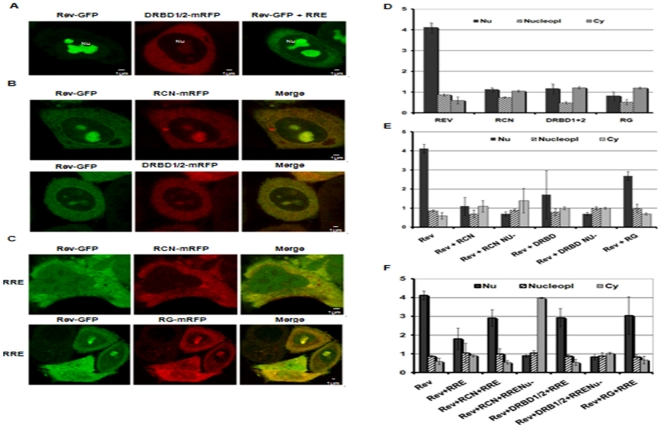
The RCN, DRBD1/2 and RG domains alter the subcellular localization of the HIV-1 Rev protein. A) Subcellular localization of Rev in cells transfected with pRev-GFP alone (left panel) or with pRev-GFP in the presence of the RRE (right panel), and subcellular localization of pDRBD1/2-mRFP alone (middle panel). The cells were fixed 24 h post-transfection and the fluorescence (green or red respectively from Rev or DRBD1/2) was registered by confocal microscopy. Rev-GFP alone is visible exclusively in nucleoli (Nu); in the presence of RRE, Rev-GFP is also visible in the cytoplasm; pDRBD1/2-mRFP is visible in the cytoplasm and nucleolus. For RCN and RG a similar subcellular localization as for DRBD1/2 was observed (results not shown). B) Subcellular localization of Rev in the presence of RCN and DRBD1/2. HeLa cells were co-transfected with pRev-GFP, with pRCN-mRFP or with pDRBD1/2 and 24 h later, the respective fluorescence was observed. Green and red signals are illustrated alone and merged in the same cell. The yellow color in the merge indicates co-localization in nucleoli and cytoplasm. A similar result was observed for the RG domain (results not shown). C) In the presence of the RRE, RCN and RG drastically alter the subcellular localization of Rev. HeLa cells were transfected as described in B, but in addition with pCMVGag2RRE (to obtain RRE), and 24 h later the cells were observed by confocal microscopy. As illustrated for RCN-mRFP (upper panel), Rev-GFP is almost excluded from the nucleoli. At low magnification, a group of cells (lower panel) illustrates the variability of the distribution of Rev in the presence of RG and RRE. A similar result was obtained for DRBD1/2 (results not shown). D) Signal intensity was quantified in different cells as illustrated in A. GFP or red fluorescence either in the nucleolus (Nu), in the nucleoplasm (Nucleopl) or in the cytoplasm (Cy) were determined for each cell. E) Signal intensity was quantified in different cells as in B, as described in D. F) Signal intensity was quantified in different cells as in C, as described in D. As control HeLa cells were co-transfected with pRev-GFP and pmRFP, but no effect on Rev localization was detected (results not shown).

The results described above indicate that NF90ctv down-regulates expression of p24 without affecting pr55Gag expression suggesting that NF90ctv can interact with and export the RRE containing mRNAs. However, the results do not explain how these interactions could influence the processing of p24 from pr55Gag. To examine the possibility that the NF90ctv domains involved in RRE-binding might disrupt the intracellular localization and dynamics of Rev, and that the altered localization of Rev may lead to the inhibition of HIV particle production, HeLa cells were transfected with pcsRev-GFP along or with one of the three NF90ctv domains (pRCN-mRFP, pDRBD1/2-mRFP or pRG-mRFP), or with pcsRev-GFP in the presence of one of the three NF90ctv domains and pCMVGag2RRE. After 24 h, Rev-GFP localization was examined by confocal fluorescence microscopy and the distribution of the fluorescent proteins in the nucleoli (Nu), nucleoplasm (Ns) and cytoplasm (Cy) was quantified (Figure 4A to C; Graph D to F). Rev alone accumulated in nucleoli, i.e. 73% of the total cell fluorescence was in the nucleoli. This concentration measures the affinity of Rev for the nucleolus as previously demonstrated by its localization [30]. The RCN-mRFP and DRBD1/2-mRFP peptides were dispersed in the cytoplasm (Figure 4A) and accumulated in the nucleolus (respectively Cy = 30–34% and Nu = 49–53%); the RG-mRFP was distributed between cytoplasm/nucleus (respectively Cy = 48%, nucleus = 52%, Nu = 32%). Rev distribution (Graph 4A) indicates that the three NF90ctv deletions entered into the nucleus and still had a preferential affinity for the nucleolus.

An alteration of Rev sub-cellular localization was observed in cells expressing the three NF90ctv-mRFP protein domains. In the presence of RCN, Rev localized in the nucleolus in 80% of the cells and in the nucleoplasm and cytoplasm in 20% of the cells (Figure 4B; Graph 4E). In the presence of DRBD1/2, Rev was localized in the nucleolus in 60% of the cells and in the nucleoplasm and cytoplasm in 40% of the cells. In the presence of RG, Rev was localized in the nucleolus in 75% of the cells, and in the nucleoplasm and cytoplasm in 25% of the cells. Interestingly, in all cases the green and red signals co-localized and their intensity correlated, suggesting possible protein-protein interactions between Rev and the three NF90ctv RRE-binding domains.

In the presence of RRE-RNA, Rev shuttled to the cytoplasm and to the nucleolus (Figure 4A; Graph 4E). However, the localization of Rev was disrupted when Rev and one of the NF90ctv protein domains were expressed in the presence of the RRE-containing Gag mRNA. Rev and RCN showed diffuse distribution in the entire cell (Figure 4C). In 40% of the cells, Rev localized in the nucleoplasm and cytoplasm and in the 60% of the cells it localized in the nucleolus. In the presence of DRBD1/2, Rev localized in the nucleolus (50% of the cells) or in the nucleoplasm and cytoplasm (50% of the cells). In the presence the RG, Rev localized preferentially in the nucleoplasm and cytoplasm (75% of the cells), and only in 25% of the cells was Rev present in the nucleolus (Figure 4C). In all cases colocalization of both the NF90ctv and Rev proteins was observed. Thus it appears that the presence of the RRE and the NF90ctv protein induce global redistribution of Rev within the subcellular compartment.

### The dynamics of intracellular distribution of Rev are altered by NF90ctv protein

**Figure 5:**
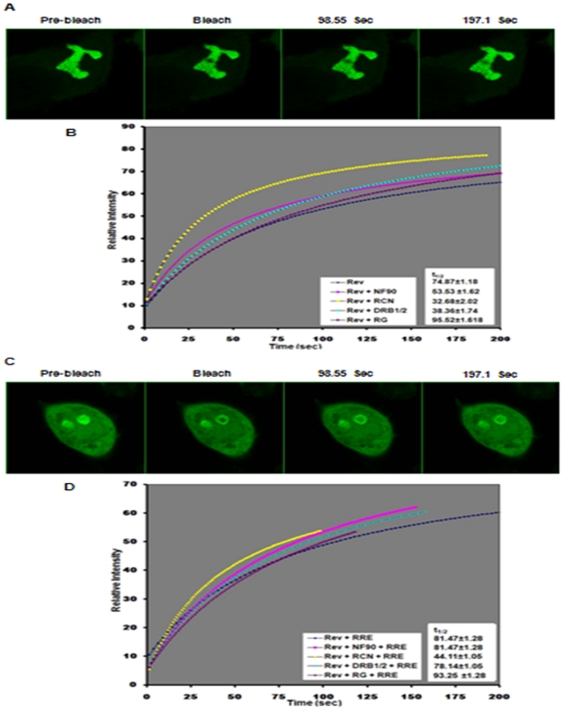
NF90ctv, RCN, DRBD1/2 and RG alter Rev mobility. A) Rev mobility was measured by FRAP in HeLa cells expressing Rev-GFP. The recovery of the Rev fluorescence in the nucleolar region bleached by laser was measured for 200 sec. Three images were taken before bleaching. B) FRAP analysis shows the effect of NF90ctv and its truncated forms on Rev mobility. HeLa cells were co-transfected with pRev-GFP in the presence of pNF90ctv-mRFP, of pRCN-mRFP, of DRBD1/2-mRFP or of pRG-mRFP and subjected to photobleaching 24 h post-transfection using a 488 nm laser. The average from at least 12 cells is shown. FRAP demonstrates that the FRAP rate of Rev-GFP alone is slower than in the presence of NF90ctv or each of its truncated forms, especially in the presence of RCN. C) The same as A but Rev is in the presence of RRE. D) The same strategies were used as described in B, but in addition, the cells were also transfected with pCMVGag2RRE. The FRAP analysis shows that in the presence of RRE and NF90ctv or each of the truncated forms of NF90ctv, the mobility of Rev was slower during the first 30 sec, but that afterwards Rev its mobility was faster, especially with RCN. As control HeLa cells co-transfected with pRev-GFP and pmRFP were used, but no effect on Rev mobility was detected (results not shown).

As discussed above, the NF90ctv protein domains (RCN, DRBD1/2 and RG) alter the sub-cellular localization of Rev. We next asked if the co-localization of Rev and the NF90ctv domains could alter the dynamics of Rev localization within the nucleus. To examine this possibility, the dynamics of Rev-GFP was measured in cells expressing Rev and specific domains of NF90ctv using FRAP. FRAP was performed using the 488 nm laser line to bleach a 0.5 µm^2^ area of the GFP signals in the nucleolus (Figure 5A). Three images were collected before bleaching, and immediately after bleaching. The recovery curves (Figure 5B) were fitted by a single exponential curve and the t_1/2_ (half-time to reach the plateau) was calculated as described previously [31]. The t_1/2_ measures the mobility of GFP-tagged proteins replacing the bleached proteins in a 0.5 µm^2^ area. In the nucleolus, the t_1/2_ recovery of Rev-GFP is 74.87±1.68 sec indicates that Rev has a relatively weak mobility due to its binding affinity for nucleolar factors (Figure 5A). By comparison the t_1/2_ recovery of NF90-GFP is 4.42 sec under the same conditions (results not shown). The t_1/2_ of the Rev-GFP co-expressed with NF90ctv or its specific domains was faster except with the RG peptide. The t_1/2_ of Rev-GFP in the presence of NF90ctv-mRFP was 53.53±1.62 sec. Moreover, the mobility of Rev varied in the presence of the different domains of NF90: it was 32.68±2.02 and 38.36±1.74 sec with RCN and DRBD1/2, respectively, and 95.52±1.61 sec with the RG-rich domain. Thus, NF90ctv and its specific domains, RCN and DRBD1/2 increase the mobility of Rev in the nucleolus; in contrast, RG decreases the mobility.

### The dynamics of Rev relocalization is faster in the presence of RRE and the NF90ctv RNA-binding domains

The impact of RRE on the dynamics of Rev localization was measured in the presence of NF90ctv protein domains that bind to and export the RRE-containing mRNA.

The experimental system consisted of HeLa cells expressing Rev-GFP and pCMVGag2RRE (which provides the RRE-containing Gag-mRNA, Figure 5C), in addition to co-transfection with expression vectors for NF90ctv, the RCN, DRBD1/2 or RG domains (Figure 5D). The dynamics of Rev distribution was evaluated by FRAP. The cells selected for this evaluation had to meet the following conditions: i) both the GFP and mRFP fluorescent proteins that were expressed as fusion proteins with Rev or the NF90ctv protein domains had to be expressed, and ii) in the presence of RRE Rev shuttles from nucleolus to the cytoplasm. We examined the dynamics of Rev localized in the nucleolus and compared its mobility in the presence or absence of RRE.

In cells expressing Rev and RRE containing Gag-mRNA, the time of Rev residency in the nucleolus increased: 81.47±1.28 sec versus 74.87±1.18 sec. (Figure 5D) suggesting that when these RNA-protein complexes are present, other cell factors are recruited to the export complex decreasing Rev mobility. Similarly, when RRE-containing Gag-mRNAs were expressed together with Rev and NF90ctv, the time of residency of Rev in the nucleolus also increased compared to Rev and NF90 without the RRE RNA: 77.92±0.95 sec versus 53.53±1.62 sec. The same effect was observed with each NF90ctv domain except RG: 44.11±1.05 versus 32.68±2.02 with RCN, 78.14±1.05 versus 38.36±1.74 with DRBD1/2 and 93.25±1.28 versus 95.52±1.61 with RG. The results show that in the presence of RRE and NF90ctv or its specific protein domains (except RG), the mobility of Rev is decreased, indicating that the Rev-export complex possibly does not use the same host components. In contrast, in the presence of the RG-rich domain, the mobility of Rev is low suggesting that different host factor(s) could be recruiting the RNA-export complex.

## Discussion

Recent attention on the significance of the NF90 family of proteins results from their varied roles in nucleocytoplasmic transport, and stimulation of antiviral response. Using CAT reporter gene assays, we previously showed that NF90ctv affects the export function of Rev [6] and its consequent effect on HIV-1 replication [17]. The results described here indicate that NF90ctv and particularly, its RCN, DRBD1/2 and RG domains affect HIV-1 particle production. Indeed, these proteins decrease the viral protein p24 both in the culture supernatants of HeLa cells transfected with the pNL4-3 provial clone and in the cell lysates (Figure 3A and 3B). The effect of NF90ctv on p24 is dose-dependent. In marked contrast, the effect of NF90ctv on intracellular pr55Gag expression was minimal. A similar result was observed using pHIVÄEnv-GFP (Figure 2). Since it was recently demonstrated that HIV-1 Gag assembly and budding are regulated by the nuclear export mechanism of the Gag-encoding mRNA, it was felt that alterations in the sub-cellular localization and the dynamics of Rev may modulate or affect Gag assembly. Two strategies were used to test this possibility: 1) Rev was expressed alone or in the presence of the three NF90ctv derivatives; 2) Rev was expressed in the presence of Gag mRNA linked to RRE alone or together with NF90ctv or its three derivatives. The dynamics of Rev localization was studied by confocal microscopy and FRAP. The presence of RCN, DRBD1/2 or RG alone or together with RRE, strongly modified the sub-cellular localization of Rev.

It has been reported that localization of Rev in the nucleolus is important for Rev function [30], [32]. In the present study, we demonstrate that the RCN, DRBD1/2 and RG regions of NF90cv induce redistribution of Rev both in the nucleus and in the cytoplasm. However, when in addition to the NF90ctv domains, RRE-RNA is also present in the cells, Rev concentrates neither in the nucleolus nor in the cytoplasm, but diffuses throughout the cell (Figure 4C). In all cases, Rev co-localizes with the NF90ctv domains, suggesting protein-protein interaction could directly or indirectly interfere with intracellular Rev trafficking.

The FRAP assays used to examine the mobility of Rev show that Rev has rather slow dynamics (T_1/2_ = 74.87±1.6 sec) in agreement with a previous report [33], perhaps related to the strong affinity of Rev binding sites within the nucleolus. This relatively long T_1/2_ could also be related to the multimerization of the Rev protein. Indeed, the three NF90ctv protein domains disrupt the sub-cellular localization of Rev in the presence or absence of RRE. We therefore utilized FRAP assays to determine the effect of the NF90ctv domains on the dynamics of Rev in the nucleolus. In the presence of NF90ctv or any of its three domains, either alone or together with RRE RNA, the mobility of Rev was changed, suggesting an effect on the affinity of Rev for its natural target, RRE. These data therefore suggest that alterations in either Rev sub-cellular localization or in the dynamics of the nucleolus or in both, could explain the effect of NF90ctv and its three domains on HIV-1 particle production (Figure 3). To our knowledge, a possible connection between the localization and dynamics of Rev with a possible negative effect on Gag assembly or budding of the virus (via down-regulation of p24), mediated by (a) cellular protein(s) or specific protein domains has so far not been reported.

Jin *et al*., [25], suggested that efficient membrane targeting by the HIV matrix (MA), requires Rev-dependent trafficking. The authors show that in the absence of Rev-dependent trafficking, the MA exhibits an inhibitory effect on Gag assembly [25]. It was previously demonstrated that perturbation of the RNA export elements of avian leucosis virus is associated with budding and genome packaging [34]. Thus disruption of both Rev localization and Rev dynamics could also affect Gag assembly. Our results support a model whereby in addition to its function in the export of singly spliced and unspliced HIV-1 transcripts, Rev may participate in other crucial steps of the HIV-1 replication cycle such as Gag assembly, packaging and budding. This interpretation of the data is in agreement with the results reported by Swanson et al., [24] who suggested that RNA export and capsid assembly are linked.

Alterations of these processes remarkably affect the HIV-1 cycle. For example, Staufen I, another double-strand specific RNA-binding protein similar to NF90, enhances pr55Gag multimerization and virus-like particle production [35]. The data presented here suggest that alteration of Rev trafficking can also regulate the functions of the HIV-1 proteins involved in assembly or budding. Together with the reports on the assembly-deficient Rev-dependent HIV-1 Gag observed in murine cells [36]–[38], our model can contribute to the study of HIV-1 Gag assembly and can help determine if there is a temporal and spatial link between Gag assembly and genome packaging.

The export of HIV-1 transcripts by Rev is highly regulated and coordinated by interaction with host factors, and in addition a link between RNA export and Gag trafficking to the plasma membrane has been described [24]. Thus, specific RNA localization contributes to protein functions at diverse levels [39]. Rev may therefore be the viral protein responsible for regulating trafficking of the unspliced genome to the packaging site. The alteration of both the localization and the dynamics of Rev, could affect the cytosolic localization of the RNA genome, resulting in differences in the composition of the ribonucleoproteins exported. If the dynamics of Rev with or without RRE is slow, but is enhanced in the presence of NF90ctv or its specific domains, this could explain coordinated changes in Rev localization. In addition, an interaction between the two proteins could affect the addition or removal of “natural” partners of Rev involved in export complex formation. This possibility is supported by the accumulation of Rev-GFP observed on the nuclear pore complexes in the presence of NF90ctv deletions leading to GFP accumulation that appears as rings in the nuclear envelope (Figure 4B). In addition, colocalization of both proteins is observed in the cytoplasm and in the nucleolus. Daelemans et al. [40] showed that Rev multimerizes in the nucleolus and this may be important in nucleocytoplasmic transport. Thus, multimerization could be altered by NF90ctv deletions; in addition, interaction between Rev and NF90ctv has been described [6].

We also show that NF90ctv has a similar function to that of HIV-1 Rev suggesting that NF90ctv can bind and export RRE-containing mRNAs to the cytoplasm where they are translated (Figure 1B). Interestingly, our results show that to bind to and export RRE, both DRBDs (DRBD1/2) must act together. Several authors have demonstrated that NF90 can bind to highly structured RNAs, such as Human adenovirus RNA_II_ [15] or the HIV Tar structure [5]. We have mapped two additional dsRNA-binding domains in NF90ctv, one in the N-Ter which includes an RCN, and one that corresponds to the last 70 amino acids (aa) in the C-Ter which is rich in RG. Surprisingly, the full-length N-Ter or C-Ter was unable to bind the RRE, which could result from conformational changes in both regions. However, the C-Ter of NF90ctv can bind to dsRNA and ssRNA synthesized *in vitro* [41]. Bearing in mind that proteins with an RG motif are involved in RNA binding, it has been proposed that the last 70 aa of the C-Ter can bind RNA. Indeed, we show here that this region is not only able to bind the RRE-containing mRNA, but is able to export the mRNA that is then translated. While the physiological relevance of the interaction of these two domains (RCN and RG) of NF90ctv with the RRE remains to be elucidated, our results suggest that one function could be interaction with exogenous dsRNA, such as the virus genome, and participation in virus replication. It will be very interesting to examine if export of unspliced mRNA mediated by NF90ctv or by its three deletions, leads to virus-like particle formation as occurs with Rev export of unspliced mRNA, or if viral genome expression in the presence of these proteins leads to the production of infectious virus.

## Materials and Methods

### Constructs and plasmids

The pCI-neo/NF90 construct that allows expression of NF90ctv was described previously [17]. To clone the NF90ctv gene and the sequence coding for its different deletion mutants in pmRFP (plasmid with the monomeric red fluorescent protein), the following strategy was used. The mRFP cassette was amplified from pcDNA3 (kindly provided by R. Y. Tsien, University of California, San Diego) using the 5′primer GGATCC GCGGCAGACCATGGCTAGCA and the 3′primer GCGGCCGC TTAGGCGCCGGTGGAGTG. The 5′primer presents a *BamH*I site (underlined) and the 3′primer a *Not*I site (underlined). pEGF-N1 (Clontech, USA), which expresses the green fluorescent protein (GFP), was cleaved with *BamH*I and *Not*I to delete the GFP cassette, and replace it by the mRFP PCR product to obtain the pmRFP construct used to transform *Escherichia coli* DH5á. The NF90ctv gene and the regions coding for its deletions were amplified from pCI-neo/NF90 using specific primers (Table 1). The forward primer contains an *EcoR*I site (underlined) and the reverse primer contains a *Sma*I site (underlined). The PCR products were digested with *EcoR*I/*Sma*I and ligated into pmRFP previously digested with the same enzymes to obtain NF90ctv and the different deletions cloned in pmRFP (Figure 1A).

**Table 1:** Primer used to cloned NF90ctv and each of its regions on pmRFP-N1.

Primers	Sequence
3′NF90nostopmRFP	ggggggCCCGGG GAAAACCTGTGTAGCCTGC
5′pAEGFPNF90Nter	GgggggGAATTC GCCCACCACTAATGCGTCCAATGCGA
3′N-terNF90mRFP	GgggggCCCGGG CTGCCTTCTCCTCTTTCAA
5′C-terNF90mRFP	ggggggGAATTC GCCCACCACTAATGAATGCCCTGATG
3′RCN	ggggggCCCGGGGTTCTGGGCTTCTTCTTAC
5′RCN	ggggggGAATTC GCCCACCACTAATGTGTCGGGGAGTG
3′DRBD1	ggggggCCCGGG CCATGTCCTGTAACACCTT
3′DRBD2	ggggggCCCGGG AAAGCTTTTCTAGGGCAGC
5′DRBD2	ggggggGAATTC GCCCACCACTAATGAACCCAGTCATG
5′RG	ggggggGAATTC GCCCACCACTAATGTTCCCTGACACC

The pcsRev-GFP construct was kindly provided by G. Pavlakis (National Cancer Institute Frederick, MD, USA) and the pCMVGag2RRE was provided by F. Maldarelli (National Institute of Allergy and Infectious Diseases, National Institutes of Health (NIH), Bethesda, MD, USA). The construct pHIV/Δenv-GFP (kindly provided by J. He (University of Indiana, Indianapolis, USA) that contains the entire HIV-1 genome but has an insertion at nucleotide position 5950 that displaces the *env* gene in the open reading frame (ORF), resulting in inhibition of Env expression; additionally, the gene encoding the Nef protein was replaced by the sequence encoding GFP [42]. pNL-4-3GFP and pNL-4-3 were kindly provided by M. Stevenson (Program in Molecular Medicine, University of Massachusetts Medical School, Worcester, Mass, USA). The expression of each construct was evaluated by fluorescence microscopy.

### Cells lines and transient-transfection

HeLa cells and 293T cells were maintained in Dulbecco minimum essential medium (DMEM; GIBCO, Carlsbad, USA) at 37°C in 5% CO_2_. The medium was supplemented with 1% penicillin/streptomycin, 1% glutamine, 1% pyruvate (Sigma-Aldrich, St. Louis, USA) and 10% fetal bovine serum (Invitrogen). For all experiments, the HeLa cells were seeded in 6-well dishes using 3×10^5^ cells per 35 mm-diameter dishes. After 24 h the cells were transfected with the appropriate plasmid DNA, using Superfect Transfection Reagent (Qiagen, USA), according to the manufacturer's instructions.

### Cell localization

To determine if NF90ctv or its truncated forms influence the cellular localization of Rev, in the presence or absence of RRE, the following strategy was used. HeLa cells were co-transfected with pcsRev-GFP either alone or in the presence of pNF90ctv-mRFP or one of the following deletion constructs (pRCN-mRFP, pDRBD1/2-mRFP or pRG-mRFP), or pRev-GFP + pmRFP as negative control. In parallel, cells were also co-transfected as described above but in the presence of pCMVGag2RRE; pCMVGag2RRE+pmRFP was used as negative control. In all assays, after 24 h the cells were treated as described previously [6]. In the nucleolus, nucleoplasm and cytoplasm of 7–10 cells of each category, the area, integrated density and mean gray value were measured using ImageJ.

### RRE-RNA binding study

To characterize the NF90ctv region(s) involved in RRE-RNA interaction, HeLa cells were co-transfected with each deletion mutant in the presence of pCMVGag2RRE that produces RRE-containing Gag mRNA. The cells co-transfected with pCMVGag2RRE+pcsRev-GFP were used as positive control. After 24 h, the cells were harvested and lysed as described [6]. The protein concentration was determined using the Bradford assay (Bio-Rad, USA). The ability of NF90ctv and its deletions to bind RRE was determined by Western blot of the expression of Gag, using HIV-1IIIB p55 Gag antibodies (obtained through the NIH AIDS Research and Reference Reagent Program, Division of AIDS, NIAID, NIH). All assays were repeated three times.

### LMB treatment

To determine if the interaction between the NF90ctv derivatives and RRE-RNA is LMB-dependent, the following strategy was used. HeLa cells were co-transfected independently with the plasmid expressing each of the regions of interest of NF90ctv in the presence of pCMVGag2RRE and incubated at 37°C in 5% CO_2_; three wells for each construct were used. After 8 h, the cells of the first well were harvested, lysed and the total extracts were maintained at −20°C; 12 ng/ml of LMB were added to the second well, and the third well served as control; both the second and third wells were incubated for an additional 4 h at 37°C in 5% CO_2_. Finally, the cells were harvested and the level of expression of Gag was evaluated by Western blot, using Gag antibodies. All assays were repeated three times.

### HIV replication assays in vitro: quantification of p24 in cell lysates and supernatants

HeLa and 293T cells were transfected with pHIV/Δenv-GFP or pNL-4-3 independently or together in the presence of pmRFP (as control). Cells were also co-transfected with different amounts of pNF90-mRFP, pRCN-mRFP, pDRBD1/2-mRFP or pRG-mRFP (0.25, 0.5, 1.0, 2.0 and 3.0 µg) DNA. After 24 h, an aliquot of the supernatant of each essay was taken and kept at −20°C. After 48 h the cells were harvested, lysed and the total proteins were quantified as described above; the supernatants were kept at −20°C. To evaluate the effect of NF90ctv or each of its deletions on HIV-1 replication, the level of expression of p24 was determined by Western blot in the cell pellets using the HIV-1 p24 Gag, and in the supernatants by ELISA, using the QuickTiter™ Lentivirus Titer Kit (Cell Biolabs, Inc USA) following the manufacturer's instructions. All assays were repeated three times.

### FRAP assays

HeLa cells were plated onto glass bottom microwell dishes at 3×10^5^ cells/well. After 24 h, the cells were co-transfected with the DNA expressing the protein of interest and maintained at 37°C in 5% CO_2_ using a professional hot-air blower in fresh DMEM supplemented with 10 nM HEPES pH 7.4. FRAP experiments were performed on a Leica SP2 AOBS confocal microscope (Leica Microsystem). Excitation of GFP was carried out at 488 nm laser line of an argon laser. Before photobleaching, three images were taken that were acquired every 1.31 sec. A diffraction-limited spot was photobleached by a single laser pulse of 100 ms at 100% of the beam. Recovery images were acquired every 1–4 s. The average intensity was determined in the photobleached region, before photobleaching and post-photobleaching. The data were normalized as was described by Negi and Olson [43].

### Antibodies

NF90ctv was detected by Western blot using Ab78 polyclonal antibodies kindly provided by JC Larcher (Université Pierre et Marie Curie, Paris, France). pAb HIV1 p24 ab63913-100 (Abcam, USA), HIV-1IIIB p55 Gag (obtained through the NIH AIDS Research and Reference Reagent Program, Division of AIDS, NIAID, NIH), β-Actin clone AC-74 (Sigma-Aldrich, St Louis, USA) was used. The GFP monoclonal antibody (1A5): sc-101536, and the β-Tubulin antibody (D-10): sc-5274 were obtained from Santa Cruz international (USA).

### Western blots

The cells were lysed with 150 ml of Kit luciferase lysis buffer (Tropix, Applied Biosystems. Fostre City, CA, USA) and 25 ml of protease inhibitor Complete EDTA-Free (Roche, Diagnostic GMBH, Mannheim, Germany), and frozen 3 times at −70°C to ensure complete cell lysis. After centrifugation, the total proteins were quantified as described [6].

### Densitometry analysis

Laser scanning (Epson Perfection® 4490 Photo) was used to convert the Western blots into digital images for subsequent densitometric analysis by the ImageJ program (http://rsbweb.nih.gov/ij/). After background correction, the integrated density of every blot and its controls (β-Tubulin and β-Actin) was measured for normalization. Finally, the Relative Intensity was calculated, dividing the absolute intensity of each band by the absolute intensity of the standards. Statistical calculations and analyses were performed with the Prism 4 (GraphPad Software, Inc) statistical software package. Student's t-test or Anova no parametric test (Kruskal-Wallis) was used to test significant differences.
